# Community Use of Antibiotics in Turkey: The Role of Knowledge, Beliefs, Attitudes, and Health Anxiety

**DOI:** 10.3390/antibiotics10101171

**Published:** 2021-09-27

**Authors:** Ümmügülsüm Gaygısız, Timo Lajunen, Esma Gaygısız

**Affiliations:** 1 Department of Anesthesia Intensive Care, Faculty of Medicine, Gazi University, 06560 Ankara, Turkey; ummugulsum@gazi.edu.tr; 2 Department of Psychology, Norwegian University of Science and Technology (NTNU), 7491 Trondheim, Norway; 3 Department of Economics, Middle East Technical University, 06800 Ankara, Turkey; esma@metu.edu.tr

**Keywords:** antibiotics, self-medication, antimicrobial resistance, community, knowledge, beliefs, attitudes, health anxiety

## Abstract

Turkey has been among the leading countries in antibiotic consumption. As a result of the 4-year National Action Plan for Rational Drug Use, antibiotic prescriptions had declined from 34.9% in 2011 to 24.6% in 2018. However, self-medication with antibiotics without prescription is common, which is not reflected in official statistics. The present study aims at investigating antibiotic use in the community and the factors related to it. A web-based survey was conducted among 945 Turkish-speaking respondents (61.3% female). The questionnaire included questions about antibiotic use for different illnesses, ways to obtain and handle leftover antibiotics, knowledge, beliefs of the antibiotic effectiveness, attitudes, health anxiety, and background factors. According to the results, 34.2% of the sample had self-medicated themselves with antibiotics without a valid prescription. The most common way to self-medicate was to use leftover antibiotics. While 80.4% knew that antibiotics are used to treat bacterial infections, 51.4% thought that antibiotics are effective for viral diseases. The most important predictor of antibiotic use frequency was the belief in their efficiency for various illnesses and symptoms, followed by negative attitudes to antibiotics, health anxiety, knowledge level, positive attitudes, and health status. The results underline the importance of targeting misbeliefs about antibiotics in future campaigns.

## 1. Introduction

Since the discovery of penicillin in 1928, antibiotics have played an enormous role in public health [1]. Today, antibiotics are the most prescribed medicines worldwide, and their consumption is rising. Between 2000 and 2015, antibiotic consumption, expressed in defined daily doses, increased 65%, and the antibiotic consumption rate increased 39%, expressed per 1000 inhabitants per day [2]. This rapid increase in antibiotic use has occurred primarily in low- and middle-income countries [2]. Due to rising incomes, availability of less expensive generic antibiotics, and lack of regulation, a further increase in antibiotic consumption is likely.

At the same time, with increased antibiotic consumption, antimicrobial resistance has become one of the biggest threats to global health [3]. Antimicrobial resistance has caused many common infectious diseases to be harder to treat and increased side effects, disability, and mortality [3,4]. Since antimicrobial resistance increases the complexity of treatment, it also leads to additional diagnostic investigations and treatments, prolonged hospital stays, and, thus, increased healthcare costs [4,5]. The World Health Organization warns that “without urgent action, we are heading for a post-antibiotic era, in which common infections and minor injuries can once again kill” [3].

Several studies have shown that antibiotic consumption rates vary significantly between different European countries, the Southern and Eastern European countries using more antibiotics than Northern and Western European countries [6,7,8,9]. Turkey has been among the leading countries in antibiotic consumption [10,11]. To address this problem, Turkey implemented an action plan for the years 2014–2017 [12], which, among other interventions, included legislation for stopping access to antibiotics without permission. The present study was conducted after the action plan was implemented, thus reflecting the attitudes and behaviours among the public after the new legislation.

Antimicrobial resistance occurs because of inappropriate and excessive use of antibiotics, in addition to poor infection control [13]. While prescription practices of antibiotics among physicians and dispensing antibiotics over the counter by pharmacists are strongly linked to regulations and the policies applied in a country [14], the general public’s use of antibiotics—especially self-medication without prescription—can be assumed to be more based on behavioural factors and characteristics of the user, such as knowledge and beliefs about antibiotics, attitudes, and personality factors. Several studies have shown that consumers have shortcomings in their knowledge about antibiotic and antimicrobial resistance and false beliefs about antibiotic effectiveness [15]. For example, a common misunderstanding is that antibiotics are effective for treating cold, flu, or other viral infections [16,17,18,19,20,21,22]. Campaigns and education programmes about the correct use of antibiotics and antimicrobial resistance can reduce inappropriate antibiotic use [23,24].

Attitudes, as enduring general evaluation, can influence the use of antibiotics. Earlier studies have addressed the attitudes to antibiotics among physicians, pharmacists, and the general population and reported that positive attitudes towards antibiotic use and interventions for reducing antimicrobial resistance could influence both prescription practices and how the general public uses antibiotics [4,25,26,27].

Earlier studies about antibiotic use in the community have mainly focused on attitudes, beliefs, and knowledge, while personality or emotions have attracted much less attention. In earlier studies, parents’ neuroticism has been found to be related to their child’s medication adherence [28]. In addition, neuroticism seems to be related to reported adherence to medication among individuals with a chronic disease [29]. It can be assumed that especially anxiety related to health might lead to higher antibiotic use.

The present study aimed to investigate the relationships between knowledge about antibiotics and antimicrobial resistance, attitudes to antibiotics and antimicrobial resistance, and health anxiety among Turkish participants.

## 2. Results

### 2.1. Antibiotic Acquisition and Use

The respondents were asked about lifetime antibiotic use; 59.7% of the respondents reported using antibiotics more than 10 times, and 38.1% reported using antibiotics less than 10 times. In terms of antibiotic use in the previous 12 months, 55.8% of the respondents reported having been using antibiotics during the previous year. As expected, antibiotics are commonly used medicines.

When asked about using antibiotics without prescription, 34.2% said that they had had antibiotics without a valid prescription. The most common way of obtaining antibiotics was via physician’s prescription (91.6%), followed by using leftover antibiotics (20.3%), from pharmacy/internet pharmacy without a doctor’s prescription an antibiotic which you used before (15.4%), from a pharmacy without a doctor’s prescription an antibiotic which the pharmacist recommended (9.7%), and from a relative or friend (11.3%). It should be noted that obtaining an antimicrobial medicine without prescription should not be possible after the 2014–2017 action plan in Turkey. When asked whom the respondents would address when ill and wishing to have antibiotics, 93.3% would turn to a physician and 6.6% to a pharmacist in the first place.

The respondents were also asked about behaviour if they felt better after 2–3 doses of antibiotics. The majority (65.1%) said they would finish the course as prescribed, while 23.0% said they would stop taking the medication and 9.7% reported that they would have a smaller dose (e.g., one tablet instead of two). When asked about the leftover antibiotics, 50.7% said that they would keep them for future use in the case of the same symptoms, 39.6% said that they would destroy the leftover medicines, and 7.5% said that they would keep the leftover medicines to use them for any other illness.

### 2.2. Knowledge and Beliefs about Antibiotics

Respondents’ knowledge of antibiotic use was measured with nine statements (see Table 1). Most of the respondents (80.4%) knew that antibiotics are used for treating bacterial infections. However, many respondents thought that antibiotics are effective for viral diseases (51.4%), fungal diseases (30.8%), and “every type of microbial infections” (48.6.%). It seems that the main confusion about antibiotics is related to viral diseases, which would also explain the widespread use in self-treatment of seasonal influenzas or the common cold.

Beliefs in antibiotic effectiveness were measured by asking respondents to evaluate the effectiveness of antibiotics in 16 different symptoms/conditions (Table 2). Antibiotics were evaluated to be the most effective for sore throat with high fever, high fever, and tooth pain. They were evaluated to be the least effective against general weakness, headache, and stomach ache. In some cases, e.g., urinary tract infection, skin infections, tooth pain, and seasonal flu, the answers were relatively equally distributed among the four response alternatives.

### 2.3. Correlates of Antibiotic Use

Pearson correlation coeffects between study variables can be seen in Table 3. Frequency of antibiotic use measured with 15 symptoms correlated statistically significantly with all predictor variables except education (Table 3). Age, subjective evaluation of health, knowledge level, and negative attitudes towards antibiotics correlated negatively with antibiotic use, knowledge (r = −0.27) and negative attitudes (r = −0.28) having the strongest correlations. These correlations indicate that older, healthier, more knowledgeable respondents with a negative attitude to antibiotics were more reserved in using antibiotics than the other respondents. Besides, beliefs about effectiveness, positive attitudes to antibiotics, and anxiety about one’s health correlated positively with antibiotic use frequency. Interestingly, these positive relationships were clearly stronger than the negative ones, which could mean that positive factors are more important factors influencing the decision to take antibiotics when ill. The correlation between belief in antibiotic efficiency for various symptoms had the strongest correlation (r = 0.60) to antibiotic use, indicating that over trust in antibiotics can increase their misuse.

In addition to correlates to antibiotic use, Table 3 also shows some other interesting correlations. Knowledge and belief in the effectiveness had a negative correlation, as expected. However, while being statistically significant, the correlation was surprisingly weak (r = −0.29), which indicates that (mis)beliefs about the effectiveness of antibiotics are influenced by correct knowledge about antibiotics only to a small degree (8% of the variance). Obviously, factors other than knowledge influence our beliefs about antibiotic efficiency. Knowledge correlated significantly with the positive attitudes to antibiotics (r = −0.38), indicating that positive attitudes to antibiotic use are partly based on lack of knowledge. The relationship between knowledge level and negative attitudes was somewhat weaker, while still statistically significant (r = 0.18). Knowledge might play a more critical role in the prevention of forming positive attitudes to antibiotic use than in negative attitudes.

As expected, anxiety about one’s health correlated negatively with positive attitudes (r = −0.11) and self-evaluated health status (r = −0.18). People who evaluated their general health low were also more anxious about health and had a more positive attitude to antibiotics. It should be noted, however, that these correlations were relatively weak, while statistically significant.

### 2.4. Predictors of Frequency of Antibiotic Use: Regression Analysis

Hierarchical multiple regression analyses were applied to model the frequency of antibiotic use in terms of the sum of 15 symptoms. In the first step, background variables (age, sex, education, health) were entered into the model. In the second step, knowledge and beliefs about effectiveness were added to the model. In the third step, two attitude scores (positive and negative attitudes) were added. The final full model also included health anxiety (see Table 4).

Models 1, 2, 3, and 4 all were statistically significant, with F-values of 6.78 (df = 4912), 93.05 (df = 6912), 77.21 (df = 8912), 69.87 (df = 9912), respectively, and the R change was also statistically significant in each step.

Table 4 shows that in the final (fourth) model, the most important predictor was the belief of effectiveness: the more effective the antibiotics were believed to be for various symptoms, the more frequently the respondents used antibiotics. Moreover, a positive attitude to antibiotics and health anxiety were positively related to antibiotic use, but to a lesser degree. Negative attitudes to antibiotics, good health, and knowledge about antibiotics were related to using fewer antibiotics, the negative attitudes being the most important predictor. Interestingly, background factors of age and education were significantly related to antibiotic use in the first step but lost their significance when the other more psychological variables, such as knowledge, beliefs, and attitudes were entered into the model. This indicates that antibiotic use among community members is primarily a behavioural issue, which can be addressed with appropriate behavioural change interventions.

## 3. Discussion

Self-medication with antibiotics without prescription is an alarming problem, especially in low- and middle-income countries [30], but also in some Southern European countries [6,7,8,9]. While governmental interventions, such as the “Rational Drug Use National Action Plan” by the Turkish government [12], play the most crucial role in regulating and monitoring antibiotic prescription practices and availability [10], targeting self-medication among community members via policy interventions is much more challenging. Self-medication with antibiotics by using leftover antibiotics, antibiotics acquired without prescription from the pharmacy, or antibiotics obtained from a friend or family member is mostly a hidden behavioural problem reflecting people’s lack of knowledge, misbeliefs, and overly positive attitude to antibiotics as a “cure for everything”. In addition to lacking knowledge, misbeliefs, and attitudes, self-medication with antibiotics can reflect an individual’s personality and emotions. For example, exaggerated worry and anxiety about one’s health can influence the adherence to medication and inappropriate use of antibiotics [29]. In the present study, knowledge and beliefs about antibiotics and antimicrobial resistance, negative and positive attitudes to antibiotic use, and health anxiety were studied as possible factors influencing antibiotic use.

In addition to an antimicrobial stewardship programme targeting hospital antibiotic use, the Turkish Ministry of Health launched a second programme (National Action Plan for Rational Drug Use 2014–2017) for curbing antimicrobial prescriptions in primary care [31]. Recent evaluations of the programme show that antibiotic prescriptions declined from 34.94% of all prescriptions in 2011 to 24.55% in 2018 [10,31]. In the present survey, however, 34.2% of respondents said that they had obtained antibiotics without a valid prescription, either from a physical or internet pharmacy or from a friend or relative. Similar rates of self-medication with antibiotics have been reported in surveys conducted, for example, in Poland (40.4%) [22] and in Jordan 41.4% [32]. The fact that more than one third of the respondents had acquired antibiotics without prescription means that inappropriate use of antibiotics cannot be controlled only by stricter prescription procedures. While being the cornerstone in the battle against antimicrobial resistance, effective information campaigns are needed in addition to policy and system-level applications. These findings are in line with the suggestion by Grigoryan et al. that interventions aimed at preventing self-medication should include public education and enforcing regulations regarding the sale of antibiotics [8].

The present study showed that the great majority (80.4%) of the respondents knew that antibiotics are used for curing bacterial diseases. However, about half (51.4%) thought that antimicrobials are used against viral diseases, and 48.6% thought that antibiotics cure every type of microbial infection. Similar findings have been reported in other studies, the proportion of people believing in antibiotic effectiveness against viruses varying from 20% to 57% [17,18,19,20]. For example, Sobek et al. reported that 35% of respondents living in Michigan, US, believed that antibiotics cure colds and flu, and 57% thought that antibiotics are good for treating viral infections [17]. Similarly, Fredericks et al. reported that over a third of Australian respondents believed that they would recover faster by taking antibiotics when suffering from a cold or flu [19]. In the present study, the misunderstanding about the use of antibiotics is reflected in answers about illnesses that antibiotics are effective for: 66.8% of the respondents thought that antibiotics are at least a little effective against seasonal flu, 64.4% similarly against the runny nose, and 85% for sore throat without fever or with low fewer. These results are in line with earlier studies, in which patients reported to have self-medicated using antibiotics primarily used for sore throat, respiratory tract infections, and influenza [22,32]. The use of antibiotics for viral infections can be based on either lacking knowledge of the aetiology of illnesses or misunderstanding of the effects of antibiotics. Whichever the reason for the misuse of antibiotics, these findings show that self-medication with antibiotics for the common cold, influenzas, and upper respiratory tract infections is the most common form of antibiotic misuse. Educational interventions and campaigns targeting especially the misuse of antibiotics for viral illnesses should be prioritised [23,24]. In an experimental study conducted in Malaysia, a short (15 min) educational session by the pharmacists improved participants’ knowledge and perception towards antibiotic use and knowledge towards antibiotic resistance [23]. Similarly, Thorpe et al. demonstrated in their experimental study conducted in the UK that providing antibiotic information substantially diminishes inappropriate expectations of antibiotics [24]. Similar short interventions by the pharmacists could be integrated to the policies in Turkey.

The regression analysis results of the present study show that the belief in antibiotic effectiveness is the strongest predictor of antibiotic use, followed by negative attitudes, positive attitudes, and health anxiety. The importance of beliefs, attitudes, and knowledge has been demonstrated earlier in several studies conducted, for example, in Australia [19], Ethiopia [21], Malaysia [23], Poland [22], and in various European countries [8]. In addition, these studies indicate that beliefs, attitudes, and knowledge are interrelated: correct beliefs require a certain level of knowledge, and attitudes are formed according to the beliefs. Therefore, all these components should be addressed in educational interventions. On the other hand, it is important to note that, in our study, beliefs were much more critical for the decision to take antibiotics than knowledge or attitudes. This might indicate that providing information about bacterial and viral infections is a less effective strategy than targeting misbeliefs about antibiotics in self-medication of common illnesses, such as common cold, influenzas, and respiratory tract infections. The public’s knowledge about basic biology, such as the difference between bacteria, viruses, and fungi, can be estimated to be very low. Therefore, the campaigns should not focus on the aetiology of illnesses but rather focus on the main misbeliefs related to the role of antibiotics in curing illnesses.

Similarly, antimicrobial resistance is an abstract concept and should not be explained at the community level, but as a real risk for an individual [26]. Besides, the results showed that negative opinions about antibiotic use had a stronger relationship to antibiotic use than positive attitudes, which is a new finding when compared to earlier studies, in which attitudes have been seen as a unidimensional continuum from negative to positive. This finding might indicate that further campaigns should rather try to implant and enforce negative attitudes to reckless antibiotic use instead of trying to change the positive attitudes. One effective strategy might be to focus on the adverse side effects of excessive antibiotics and the effects of antimicrobial resistance.

Finally, health anxiety predicted antibiotic use frequency significantly. This means that individuals with health-related anxiety may use antibiotics as a precaution in the case when the aetiology of the illness, e.g., sore throat or fever, is not clear. In this way, the use of medicines in general and antibiotics, in this case, might be used to control health-related anxiety. As demonstrated with medication for chronic illnesses, personality (e.g., neuroticism) and patients’ emotional states can influence the adherence to medication [29]. While adherence to medication in Axelsson et al. [29] refers to individual (personality) differences in medication adherence in chronic diseases, the same findings might also apply to antibiotic use. Patients with certain personality structure, such as high anxiety related to health, may be much more likely to self-medicate with antibiotics. For some reason, individual difference factors, such as personality or emotionality, have attracted very little attention among researchers studying antibiotic use.

This study has some limitations which should be taken into account. Firstly, the sampling was based on convenience sampling among students and their relatives, friends, and acquaintances. Thus, the sample does not represent the Turkish population in terms of age and education level. It should be noted, however, that collecting representative samples in Turkey is difficult, because the response rate to postal surveys tends to be very low, which compromises the representativeness of the sample. Household or telephone interviews could be an alternative, but those methods are also vulnerable to sampling bias due to the lack of anonymity. The present study was conducted by sending invitations to students and using anonymous internet sampling, which increases the anonymity of the answers. In addition, the respondents’ socio-economic status or place of residence (urban or rural) was not asked, although these factors might influence self-medication with antibiotics. It should be noted, however, that the results in the present study are in line with studies conducted in other countries. In future studies, age- and region-stratified representative samples should be collected to further study antibiotic use in communities in Turkey.

## 4. Materials and Methods

### 4.1. Data Collection and Participants

The data were collected by advertising the web link to SurveyMonkey among students at the Middle East Technical University, Ankara, Turkey. The students and their friends and relatives were invited to fill in an online survey. The online questionnaire contained information on the background to the study, objectives, voluntary nature of participation, declarations of anonymity and the confidentiality of all data, and informed consent was obtained from all participants. Norwegian Centre for Research Data (NSD) was consulted to see if ethical permission is needed for the study. Since the study was conducted on the internet and no identification data were collected, the survey does not contain any sensitive data as defined by NSD, so no ethical permission was required.

The data included 945 Turkish speaking respondents, of which 61.3% were female. The respondents were fairly young (M = 26.4, SD = 10.2 years) compared to the median age of the population of Turkey (31.5 years), which is understandable, since the sampling was conducted among university students and people related to them. The sample was also more educated than the population on average: 18.6% of the respondents were high school graduates and 77.8% were university graduates or university students, while only 3.6% of the respondents had less than a high school degree. When asked about self-assessed general health, 0.4% evaluated their health “bad”, 1.6% “rather bad”, 10.3% “neither bad nor good”, 41.3% “rather good”, and 46.3% “good”.

### 4.2. Questionnaire

The survey questions were constructed by modifying questions used in literature, brainstorming based on clinical experiences, and experiences from our earlier studies.

#### 4.2.1. Ways to Obtain, Use, and Handle Leftover Antibiotics

The respondents were asked, with five alternatives, how they usually get their antibiotics. The options were “From pharmacy with a doctor’s prescription”, “From pharmacy/internet pharmacy without a doctor’s prescription an antibiotic which you used before”, “From pharmacy without a doctor’s prescription an antibiotic which the pharmacist recommended”, “Use antibiotic which was leftover from an earlier prescription”, and "From a friend or a family member”. The respondents could choose as many alternatives as they wished.

The respondents were also asked to indicate if they turned to a physician (antibiotic with prescription) or to a pharmacist (antibiotic without prescription) in the first place to get antibiotics. In terms of completing the treatment, the respondents were asked what they would do if they felt better after 2–3 doses of antibiotic treatment. The answer alternatives were “stop taking medicine to avoid unnecessary use”, “continue the course but with a smaller dose than prescribed”, and “continue the course to end as prescribed”. The respondents were also asked about handling the leftover antibiotics. The three answer alternatives were “I would destroy the leftover antibiotics”, “I would keep them for future use in the case of the same illness”, and “I would keep them for future use for any illnesses”.

#### 4.2.2. Antibiotic Use

The dependent variable in this study was the frequency of antibiotic use for 15 illnesses or symptoms (see Table 1). The respondents indicated how often they use antibiotics for the listed 15 illnesses with a 5-point frequency scale from “never” to “every time”. Answers to the questions were averaged so that the scores could range from 1 to 5. The antibiotic use scale showed high internal consistency with Cronbach’s alpha reliability 0.91, indicating that antibiotic use is a generalised habit occurring for different illnesses and symptoms.

#### 4.2.3. Knowledge and Beliefs about Antibiotics

Participants’ knowledge about antibiotics was measured with a question “Which of the following are the purposes/uses of antibiotics?” about nine different purposes (see Table 1). The respondents could choose as many uses as possible. A correct choice gave respondents one point (+1), and each wrong answer one minus point (−1). The total knowledge score was calculated by extracting from the correct answer (1 point) all wrong answers chosen. Hence, the scores could range from −8 to +1, with a higher score reflecting better knowledge.

Beliefs about the effectiveness were measured with the question, “How effective (cure or shorten the illness or make it easier) are antibiotics for following illnesses or symptoms?” Respondents evaluated the effectiveness of antibiotics with a 4-point answer scale (from “not at all effective” to “very effective”) in terms of 16 illnesses (see Table 2). The total “belief in effectiveness” score was calculated by averaging the answers to the 16, so the scores could range from 1 to 4. A high score reflects a strong belief that antibiotics would be an effective treatment for the illnesses listed. The Cronbach’s alpha reliability was 0.89, indicating high internal consistency for the scale.

#### 4.2.4. Attitudes to Antibiotics

To measure attitudes to antibiotics and antibiotic resistance, they were measured with 17 statements, to which the respondents replied with a 5-point scale from “totally disagree” (1) to “totally agree”. The responses were analysed with factor analysis (principal axis factoring method with mineigen >1 criterion and promax rotation) to see if the 17 attitudes group to subfactors. The results of the factor analysis yielded two clearly interpretable factors which could be named as “positive attitudes to antibiotics” (e.g., ‘Antibiotics are safe drugs; hence they can be commonly used”; “When I get a fever, antibiotics help me to get better more quickly”; “Antibiotic resistance is a problem only in hospitals and with severely ill patients”) and “negative attitude to antibiotics” (e.g., “If taken too often, antibiotics are less likely to work in the future”; “Antibiotics can have severe side effects”; “It is good to avoid unnecessary use of antibiotics”). The reliability coefficients (Cronbach alpha) were 0.70 and 0.77 for “positive attitudes” and “negative attitudes”, respectively. The attitude scales correlated negatively with each other (r = −0.57). The altitude scores could range from 1 to 5.

#### 4.2.5. Health Anxiety Scale

Health anxiety was measured with Health Anxiety Inventory (HAE) [33]. HAE contains 14 statements about health anxiety which had four answer options. The health anxiety score was calculated by calculating the average score of the statements so that the scores could range from 1 to 4. The alpha reliability of the scale was 0.85, indicating high internal consistency.

#### 4.2.6. Background Questions

The survey included questions about age, sex, education level, and use of antibiotics during the past 12 months (answers: yes, no) and lifetime (answers: less than 10 times, 10 times or more).

### 4.3. Statistical Analysis

The data were collected with SurveyMonkey and analysed with SPSS v27. The data were analysed with descriptive and frequency analysis, factor analysis, reliability analysis, Pearson correlation coeffects, and hierarchical multiple regression analysis.

## 5. Conclusions

The present study showed that self-medication with antibiotics without prescription is still a significant problem in Turkey, while the antibiotic prescription rate among community physicians has dropped. The public’s misbeliefs about antibiotics are mostly related to the effectiveness of antibiotics in viral infections, such as influenzas and the common cold. These wrong beliefs should be targeted in future campaigns and educational interventions. In addition, the role of health anxiety should be studied further in relation to self-medication with prescription medicines in general, and antibiotics in particular. The public should be warned about the risks of self-medication “just in case”.

## Figures and Tables

**Table 1:** Answers to the question “Which of the following are the purposes/uses of antibiotics?”.

Statement	No (%)	Yes (%)
Cure bacterial diseases.	19.6	80.4
Cure viral diseases.	48.6	51.4
Cure fungal diseases.	69.2	30.8
Cure every type of microbial infection.	51.4	48.6
Reduce pain.	83.7	16.3
Strengthen the body so that we do not become ill.	89.9	10.1
Provide necessary vitamins and minerals for the body.	95.1	4.9
Reduce blood sugar level.	98.4	1.6
Reduce blood pressure.	98.1	1.9

**Table 2:** Beliefs about the effectiveness of antibiotics: distribution of answers.

Symptom/Condition	Not at All Effective (%)	A Little Effective (%)	Somewhat Effective (%)	Very Effective (%)
Runny nose	35.6	21.4	28.6	14.4
Nasal congestion	49.9	26.4	17.6	6.1
Cough	25.0	27.4	33.5	14.1
Sore throat with high fever (over 39 °C)	9.2	13.1	29.8	47.9
Sore throat without fever or low fever (up to 38 °C)	15.0	24.9	37.7	22.4
High fever	14.8	15.9	33.1	36.1
Seasonal flu	33.2	26.7	25.2	14.8
Aches and pains	42.1	24.8	22.3	10.8
Vomiting	49.2	27.1	18.1	5.6
Diarrhoea	39.3	24.4	27.6	8.8
Skin infections	28.0	20.6	31.2	20.2
Headache	60.6	18.8	14.1	6.5
Stomach ache without vomiting or diarrhoea	54.8	23.6	16.5	5.2
Weakness	59.4	21.0	15.2	4.4
Tooth pain	20.3	15.9	30.5	33.2
Urinary tract infection	27.7	22.2	27.9	22.2

**Table 3:** Correlations among study variables.

	1	2	3	4	5	6	7	8
1. Frequency of antibiotic use	1.00							
2. Age	−0.12 ***	1.00						
3. Education	−0.04	−0.23 ***	1.00					
4. Self-evaluated health status	−0.10 **	0.01	0.07 *	1.00				
5. Knowledge about antibiotics	−0.27 ***	−0.01	0.03	0.02	1.00			
6. Beliefs about antibiotic effectiveness	0.60 ***	−0.14 ***	−0.01	−0.04	−0.29 ***	1.00		
7. Positive attitudes to antibiotic use	0.34 ***	−0.09 **	−0.08 *	0.05	−0.38 ***	0.37 ***	1.00	
8. Negative attitudes to antibiotic use	−0.28 ***	0.10 **	0.04	0.06	0.18 ***	−0.18 ***	−0.37 ***	1.00
9. Health Anxiety Scale score	0.15 ***	−0.14 ***	0.06	−0.18 ***	−0.03	0.10 **	0.06	−0.11 ***

* *p* < 0.05; ** *p* < 0.01; *** *p* < 0.001.

**Table 4:** Hierarchical regression results for predicting antibiotic use.

Step		B	Std. Error	Beta	*t*	CI 95% of B
1 (r^2^ = 0.03)	(Constant)	2.90	0.22		13.08 ***	2.46; 3.33
	Age	−0.01	0.00	−0.13	−3.78 ***	−0.01; 0.00
	Sex	−0.02	0.05	−0.01	−0.45	−0.11; 0.07
	Education	−0.09	0.04	−0.07	−20.04 *	−0.17; 0.00
	Health	−0.09	0.03	−0.10	−3.10 **	−0.15; −0.03
2 (r^2^ = 0.38)	(Constant)	1.05	0.20		5.38 ***	0.67; 1.44
	Age	0.00	0.00	−0.05	−1.68	−0.01; 0.00
	Sex	0.01	0.04	0.01	0.30	−0.06; 0.08
	Education	−0.06	0.03	−0.04	−1.64	−0.12; 0.01
	Health	−0.07	0.02	−0.08	−2.95 **	−0.12; −0.02
	Knowledge	−0.06	0.01	−0.11	−4.01 ***	−0.08; −0.03
	Beliefs about effectiveness	0.62	0.03	0.56	20.24 ***	0.56; 0.68
3 (r^2^ = 0.41)	(Constant)	1.50	0.24		6.18 ***	1.03; 1.98
	Age	0.00	0.00	−0.03	−1.09	−0.01; 0.00
	Sex	0.00	0.04	0.00	−0.12	−0.07; 0.07
	Education	−0.04	0.03	−0.03	−1.15	−0.10; 0.03
	Health	−0.07	0.02	−0.08	−2.96 **	−0.12; −0.02
	Knowledge	−0.04	0.01	−0.07	−2.49 *	−0.06; −0.01
	Beliefs about effectiveness	0.58	0.03	0.52	18.44 ***	0.52; 0.64
	Positive attitudes	0.07	0.03	0.07	2.31 *	0.01; 0.13
	Negative attitudes	−0.14	0.03	−0.13	−4.73 ***	−0.20; −0.08
4 (r^2^ = 0.41)	(Constant)	1.24	0.26		4.76 ***	0.73; 1.76
	Age	0.00	0.00	−0.02	−0.78	0.00; 0.00
	Sex	−0.01	0.04	−0.01	−0.21	−0.08; 0.06
	Education	−0.04	0.03	−0.03	−1.29	−0.11; 0.02
	Health	−0.06	0.02	−0.06	−2.48 *	−0.11; −0.01
	Knowledge	−0.04	0.01	−0.07	−2.51 *	−0.06; −0.01
	Beliefs about effectiveness	0.58	0.03	0.52	18.33 ***	0.52; 0.64
	Positive attitudes	0.07	0.03	0.07	2.28 *	0.01; 0.13
	Negative attitudes	−0.13	0.03	−0.13	−4.55 ***	−0.19; −0.08
	Health Anxiety	0.11	0.04	0.07	2.65 **	0.03; 0.19

* *p* < 0.05; ** *p* < 0.01; *** *p* < 0.001.

## Data Availability

Not applicable.
